# Epigenetic silencing of *ADAMTS18* promotes cell migration and invasion of breast cancer through AKT and NF‐*κ*B signaling

**DOI:** 10.1002/cam4.1076

**Published:** 2017-05-15

**Authors:** Hongying Xu, Qian Xiao, Yu Fan, Tingxiu Xiang, Chen Li, Chunhong Li, Shuman Li, Tianli Hui, Lu Zhang, Hongzhong Li, Lili Li, Guosheng Ren

**Affiliations:** ^1^ Chongqing Key Laboratory of Molecular Oncology and EpigeneticsThe First Affiliated Hospital of Chongqing Medical UniversityChongqingChina; ^2^ Cancer Epigenetics LaboratoryDepartment of Clinical OncologyState Key Laboratory of Oncology in South China, Sir YK Pao Center for Cancer and Li Ka Shing Institute of Health SciencesCUHK Shenzhen Research InstituteThe Chinese University of Hong KongHong Kong; ^3^ Department of OncologySuining Center HospitalSuiningChina

**Keywords:** *ADAMTS18*, cancer, metastasis, methylation, tumor suppressor

## Abstract

ADAMTS18 dysregulation plays an important role in many disease processes including cancer. We previously found *ADAMTS18* as frequently methylated tumor suppressor gene (TSG) for multiple carcinomas, however, its biological functions and underlying molecular mechanisms in breast carcinogenesis remain unknown. Here, we found that *ADAMTS18* was silenced or downregulated in breast cancer cell lines. *ADAMTS18* was reduced in primary breast tumor tissues as compared with their adjacent noncancer tissues. *ADAMTS18* promoter methylation was detected in 70.8% of tumor tissues by methylation‐specific PCR, but none of the normal tissues. Demethylation treatment restored *ADAMTS18* expression in silenced breast cell lines. Ectopic expression of ADAMTS18 in breast tumor cells resulted in inhibition of cell migration and invasion. Nude mouse model further confirmed that ADAMTS18 suppressed breast cancer metastasis in vivo. Further mechanistic studies showed that ADAMTS18 suppressed epithelial‐mesenchymal transition (EMT), further inhibited migration and invasion of breast cancer cells. ADAMT18 deregulated AKT and NF‐*κ*B signaling, through inhibiting phosphorylation levels of AKT and p65. Thus, ADAMTS18 as an antimetastatic tumor suppressor antagonizes AKT and NF‐*κ*B signaling in breast tumorigenesis. Its methylation could be a potential tumor biomarker for breast cancer.

## Introduction

Breast cancer is one of the most commonly diagnosed malignant tumors and the leading cause of cancer death in women 1. Data indicate that breast cancer dissemination and distant metastasis is an early onset and inherent genetic property that does not adhere to the multistep tumorigenesis theory 2. Therefore, timely metastasis‐related biomarker testing helps to reduce breast cancer mortality.

A disintegrin and metalloproteinase with thrombospondin motifs (ADAMTS) family was first identified in 1997 3. Compared with members of the metalloproteinases (MMPs) and a disintegrin and metalloproteinases (ADAMs), ADAMTS are secreted proteases, being synthesized and secreted by a variety of cells including macrophagocyte, vascular endothelial cell, fibroblasts, and smooth muscle cells and are usually integrated with the extracellular matrix 4. Currently, 19 family members have been identified, and some members have been implicated in cancer progression, such as *ADAMTS8* 5, *ADAMTS9* 6, *ADAMTS15* 7 and so on. *ADAMTS18*, a novel member of ADAMTS family which we are focusing on, located at 16q23.1 in the human genome 8, was identified in 2002 9.

Previous studies have reported *ADAMTS18* possessing tumor‐suppressing activities, which were reversed by promoter hypermethylation in several malignancies, including nasopharyngeal, esophageal squamous cell, hepatocellular, cervical carcinomas 8. Subsequent studies have shown the prevalence of hypermethylation and low expression of *ADAMTS18* in gastric, colorectal, pancreatic, and clear cell renal cell carcinomas 10, 11. Based on these findings, we have identified *ADAMTS18* as a candidate TSG. However, studies on its roles in tumor suppression and related molecular mechanisms in breast cancer remain unclear. Here, we evaluated the expression and methylation status of *ADAMTS18* in breast cancer cell lines and primary tumors, and further assessed its biological functions. We also explored relevant mechanisms of *ADAMTS18* in breast tumorigenesis.

## Materials and Methods

### Cell lines and samples

Several breast tumor cell lines (BT549, MB231, MB468, MCF7, SK‐BR‐3, T47D, YCC‐B2, YCC‐B3, and ZR‐75‐1) were used as described previously 12, 13. All samples used in these studies were obtained from the tissue bank of the First Affiliated Hospital of Chongqing Medical University 14, including normal breast tissues and paired primary breast tumor tissues. This study was approved by the Institutional Ethics Committees of the First Affiliated Hospital of Chongqing Medical University (approval notice # 2010/2012(23)).

Cell lines were treated with 10 mmol/L 5‐aza‐2′‐deoxycytidine (Sigma‐ Aldrich, St Louis, MO) for 3 days and further treated with 100 nmol/L trichostatin A (TSA; Cayman Chemical Co., Ann Arbor, MI) for an additional 24 h.

### Establishment of stable cell lines

*ADAMTS18* full‐length cDNA‐expressing vector pcDNA3.1 (+)‐ADAMTS18 was generated as previously described and the sequence was verified 8. To establish stably transfected tumor cells with *ADAMTS18* expression, cells (BT549, MB231 and MCF7) were transfected with ADAMTS18 expression plasmids using Lipofectamine^™^ 2000 (Invitrogen), and further screened a monoclonal cell population by G418.

### Semiquantitative RT‐PCR and quantitative RT‐PCR (qRT‐PCR)

Semiquantitative RT‐PCR was performed as described previously 15. RT products were amplified with 32 cycles for *ADAMTS18*, and 23 cycles for *β*‐actin, using Go‐Taq^®^ (Promega, Madison, WI). SYBR^®^ Green Master Mix (Applied Biosystems, Grand Island, NY) was used for qPCR analyses as measured by the 7500 Real‐Time PCR System (Applied Biosystems) as described previously 16, 17.

### DNA bisulfite treatment and methylation‐specific PCR (MSP)

Genomic DNA was extracted from tumors and normal tissues using a QIAamp DNA Mini Kit (Qiagen, Hilden, Germany). DNA bisulfite treatment and methylation‐specific PCR (MSP) analysis were performed as described previously 18. Bisulfite‐treated DNA was amplified by MSP using *ADAMTS18* methylated or unmethylated primer set (Table S2) by AmpliTaq‐Gold DNA polymerase (Applied Biosystems, Foster City, CA), with an annealing temperature of 60°C for methylation detection, and 58°C for unmethylation detection.

### Cell viability assay

Cells were replated into 96‐well plates after being transfected with ADAMTS18 expression vector and empty vector for 48 h, and further measured using the Cell Counting Kit‐8 (CCK‐8, Beyotime, Shanghai, China) as described previously 17, 19.

### Colony formation assay

Monolayer culture was used for colony formation assay. Cells were selected for ~14 days with presence of G418, after transfection for 48 h. Surviving colonies (≥50 cells/colony) were counted and stained with gentian violet. All experiments were performed in triplicate wells, three times.

### Migration and invasion assays

Cell motility and invasive abilities were assessed by Transwell® and Matrigel™ invasion assays (Corning Life Sciences, Bedford, MA) as described previously 12, 13., Cells were migrated and invaded to the lower side of the membrane, and further stained with 0.1% crystal violet. Cells were then counted in five microscopic fields, and the mean values were counted.

### Wound healing assay

Wound healing assay was performed as described previously 17, 19. A mechanical wound was created after scratching with a pipette tip, and images were taken at different time points. The distance between the wound edges was measured and quantified.

### Tail vein assay of cancer metastasis

*ADAMTS18*‐expressing MB231 cells or control cells (2.5 × 10^6^ cells) were injected into the lateral tail vein of female Balb/c nude mice in a volume of 0.15 mL as described previously 20, with four mice per group (control group: #3647, #3648, #3682, and #3683; experimental group: #3649, #3684, #3685, and #3698). After 8 weeks, two groups of mice were killed, and metastatic nodules were counted on the lung surface, which were further confirmed by histopathological analysis.

### Western blot

Protein Extraction Reagent (Thermo Scientific, Rockford, IL) was used for protein extraction. Western blot analysis was performed as described previously 12, 13, 16, 19.Antibodies included: ADAMTS18 (ab135728), occludin (ab31721, Abcam, Cambridge, UK); AKT (#4691), p‐AKT (#4060), p‐P65 (#3033), E‐cadherin (#3195), vimentin (#5741), Snail (#3879, Cell Signaling Technology, Danvers, MA, USA); *β*‐actin (LK‐ab008‐100; Liankebio, China).

### Statistical analysis

Statistical analysis was performed using SPSS 16.0 software (SPSS, Chicago, IL, USA). The Wilcoxon rank‐sum test was used for evaluating *ADAMTS18* expression. Fisher's exact tests were used to analyse correlation of *ADAMTS1* methylation with clinicopathological parameters. The two‐tailed *t*‐test was used to assess migration data. Data are reported as values of means ± SD. *P *<* *0.05 was considered as statistically significant.

## Results

### *ADAMTS18* is downregulated in breast cancer cells and tissues

**Figure 1:**
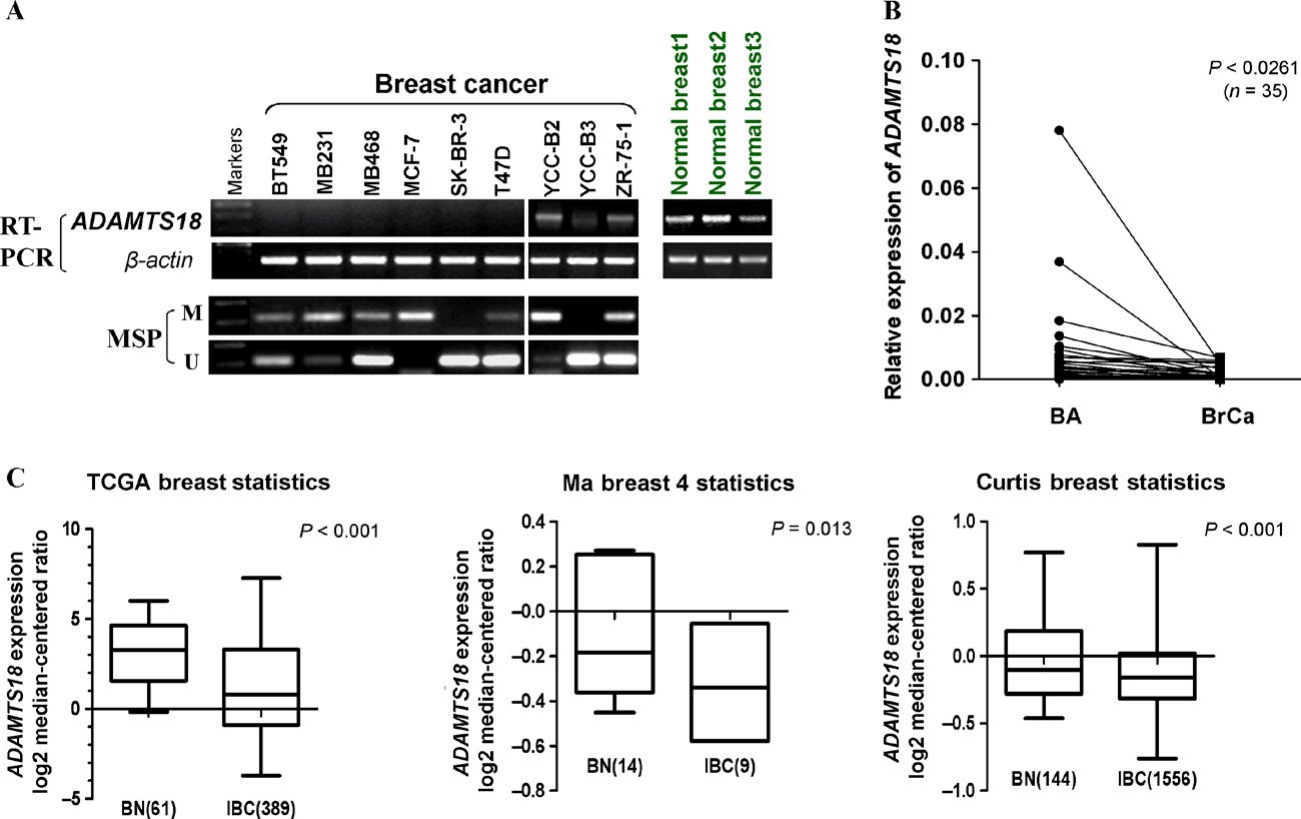
*ADAMTS18* is frequently downregulated in breast cancer cell lines and breast cancer specimens. (A) The expression profile of *ADAMTS18* mRNA in breast cancer cell lines and normal breast tissues by semiquantitative RT‐PCR, using *β*‐actin expression as an internal control. (B) *ADAMTS18* mRNA levels were significantly decreased in primary breast cancer as compared with adjacent noncancer tissues as determined by quantitative real‐time PCR (*n* = 35). *ADAMTS18* mRNA levels were normalized with the *β*‐actin mRNA level. (*P *<* *0.05). (C) *ADAMTS18* mRNA expression levels was assayed from the Oncomine database tool (http://www.oncomine.org). BN, Normal Breast tissues; BrCa, breast cancer; IBC, Invasive Breast Carcinoma.

Initial evidence has indicated that *ADAMTS18* gene expression is decreased in many human cancer cells 8, 10. We first evaluated *ADAMTS18* expression in a panel of breast cancer cell lines and normal breast tissues using semiquantitative RT‐PCR. We found that *ADAMTS18* was readily expressed in normal breast tissues, but frequently silenced or decreased in all nine breast tumor cell lines studied (Fig. 1A). *ADAMTS18* expression in primary breast tumors and surgical‐margin tissues was further examined by qRT‐PCR. Results showed that *ADAMTS18* was obviously decreased (31/35 of the samples) in breast cancer tissues, as compared with surgical‐margin tissues (*P *<* *0.05) (Fig. 1B). Moreover, through analyzing the online Oncomine database, *ADAMTS18* was significantly downregulated in breast cancer tissues, compared with normal breast tissues (Fig. 1C).

### Promoter methylation contributes to *ADAMTS18* suppression in breast cancer

**Figure 2:**
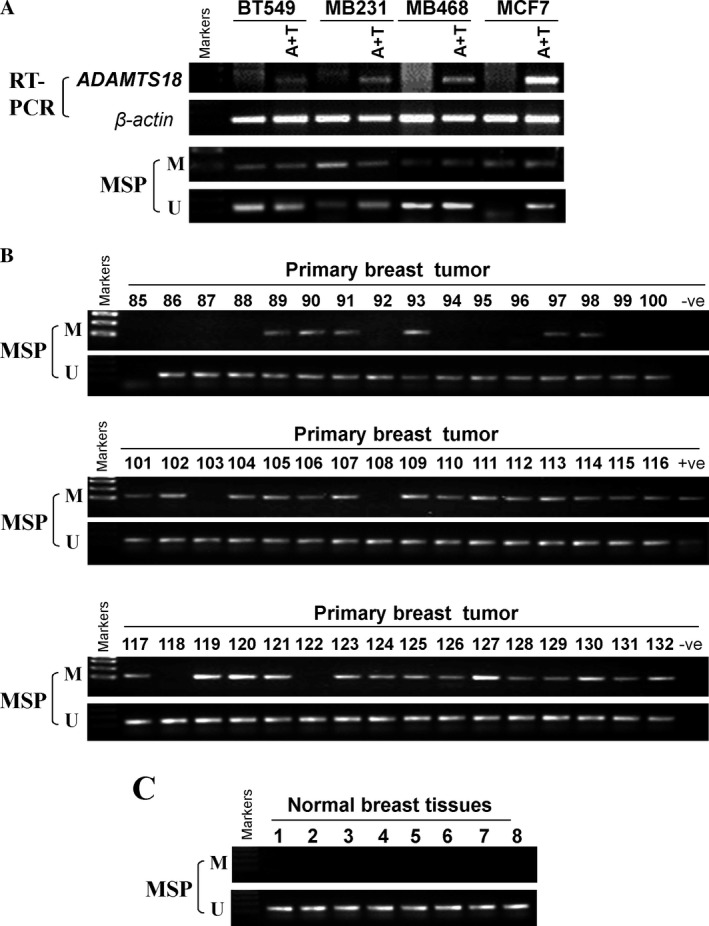
Promoter methylation of *ADAMTS18* in breast cancer. (A) *ADAMTS18* mRNA expression was restored after treatment with demethylation reagent, Aza and histone deacetylase inhibitor, TSA in four breast cancer cell lines (A+T refers to addition of Aza and TSA in culture media). Demethylation was confirmed by MSP (methylation‐specific PCR). (B) Promoter methylation of *ADAMTS18* in primary breast cancer was determined. (C) MSP analysis of *ADAMTS18* methylation in normal tissues. M, methylated DNA; U, unmethylated DNA.

We next examined whether promoter methylation leads to *ADAMTS18* suppression in breast cancer. MSP showed that *ADAMTS18* was methylated in breast tumor cell lines with its silencing or downregulation (Fig. 1A). We further evaluated whether promoter methylation directly regulates *ADAMTS18* gene expression. We treated four cell lines (BT549, MB231, MB468, and MCF7) with demethylating agent Aza and histone deacetylase inhibitor TSA. After treatment, *ADAMTS18* expression was restored to different degrees. Meanwhile, MSP showed that the CGI (CpG island) was demethylated to some extent in the presence of drug (Fig. 2A). These results suggest that promoter methylation mechanism is responsible for *ADAMTS18* silencing in breast cancer cells.

To further evaluate *ADAMTS18* promoter methylation in primary breast tumors, we examined *ADAMTS18* methylation in 48 primary breast tumor samples and eight normal breast tissue samples. *ADAMTS18* methylation was observed in 34 out of 48 (70.8%) primary tumors, but not in normal breast tissues (Fig. 2B, C and Table 1), indicating methylation‐mediated *ADAMTS18* inactivation in breast cancer. We further analyzed the association between *ADAMTS18* methylation and clinicopathological features, including age, tumor size, tumor grade, lymph node metastasis, ER status, PR status, HER2 status, P53 status, and Ki67 status. No significant correlation of *ADAMTS18* methylation with any clinicopathological features (Table S1) was observed.

**Table 1:** Promoter methylation status of *ADAMTS18* in primary breast tumors

Tissue	Samples (number)	*ADAMTS18* promoter		Frequency of methylation	*P* value
		methylated	Unmethylated		
Breast tumor	48	34	14	70.8%	0.0002
Normal breast	8	0	8	0%	

### ADAMTS18 is an antimetastatic tumor suppressor for breast cancer cells

**Figure 3:**
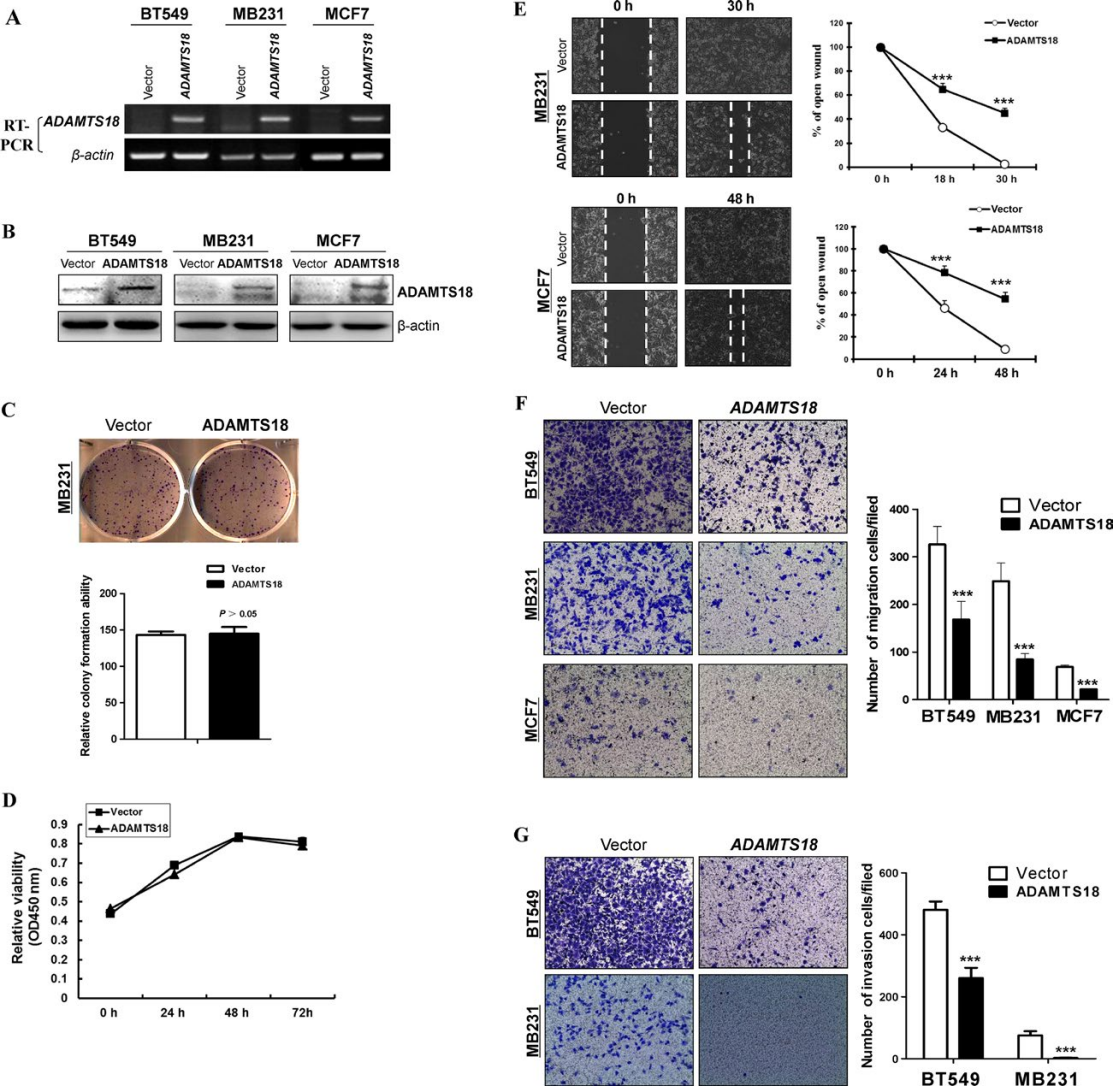
Effects of ectopic ADAMTS18 on migration and invasion of breast cancer cells. (A) RT‐PCR analyses of *ADAMTS18* mRNA expression in three *ADAMTS18* overexpressing cell lines (BT549, MB231, and MCF7). (B) Western blotting analyses of ADAMTS18 expression in *ADAMTS18* overexpressing BT549, MB231, and MCF7 cells. (C) Representative colony formation assay and quantitative analysis. The numbers of G418‐resistant colonies in vector‐transfected controls were set to 100%, values are expressed as the mean ± SD from three experiments. (D) CCK‐8 assay for cell proliferation on vector‐ and ADAMTS18‐transfected MB231 cells. (E) Migration of *ADAMTS18*‐transfected or empty vector‐transfected tumor cells by scratch wound healing assays. The width of the remaining open wound was measured in relation to time 0 h. Original magnification, 100×. *P <* *0.05, **P <* *0.01, and ***P <* *0.001. (F) Significant reduction in cell migration was observed in cells expressing *ADAMTS18* compared with control cells. Representative images of migrated BT549, MB231, and MCF7 cells in each group are shown. Migrating cells at the lower surface of the Transwell^®^ chamber were counted. (E) Effects of ADAMTS18 overexpression on invasive ability of BT549 and MB231 cells by the Boyden chamber invasion assay. (G) Cells at the lower surface of the Transwell^®^ chamber were counted. Original magnification, 100×. *P <* *0.05, **P <* *0.01, and ***P <* *0.001.

We further assessed tumor‐suppressive functions of ADAMTS18. RT‐PCR and western blot confirmed ADAMTS18 expression in *ADAMTS18*‐transfected breast tumor cells (Fig. 3A, B). Colony formation and CCK8 assays showed that overexpression of *ADAMTS18* had no detectable effect on the proliferation of breast cancer MB231 cells (*P *>* *0.05) (Fig. 3C, D).

Scratch wound healing assays showed that *ADAMTS18*‐transfected cells showed slower closure of the scratched wound in MB231 and MCF7 cell lines (*P *<* *0.001) (Fig. 3E). In addition, we measured cell migration and invasion using Transwell^®^ assays (with or without Matrigel) in *ADAMTS18‐*transfected and the respective control cells. Results showed that *ADAMTS18* overexpression significantly decreased cell migration in BT549, MB231, MCF7, and invasion in BT549 and MB231 (*P *<* *0.001), confirming the antimetastatic properties of *ADAMTS18* (Fig. 3F, G).

**Figure 4:**
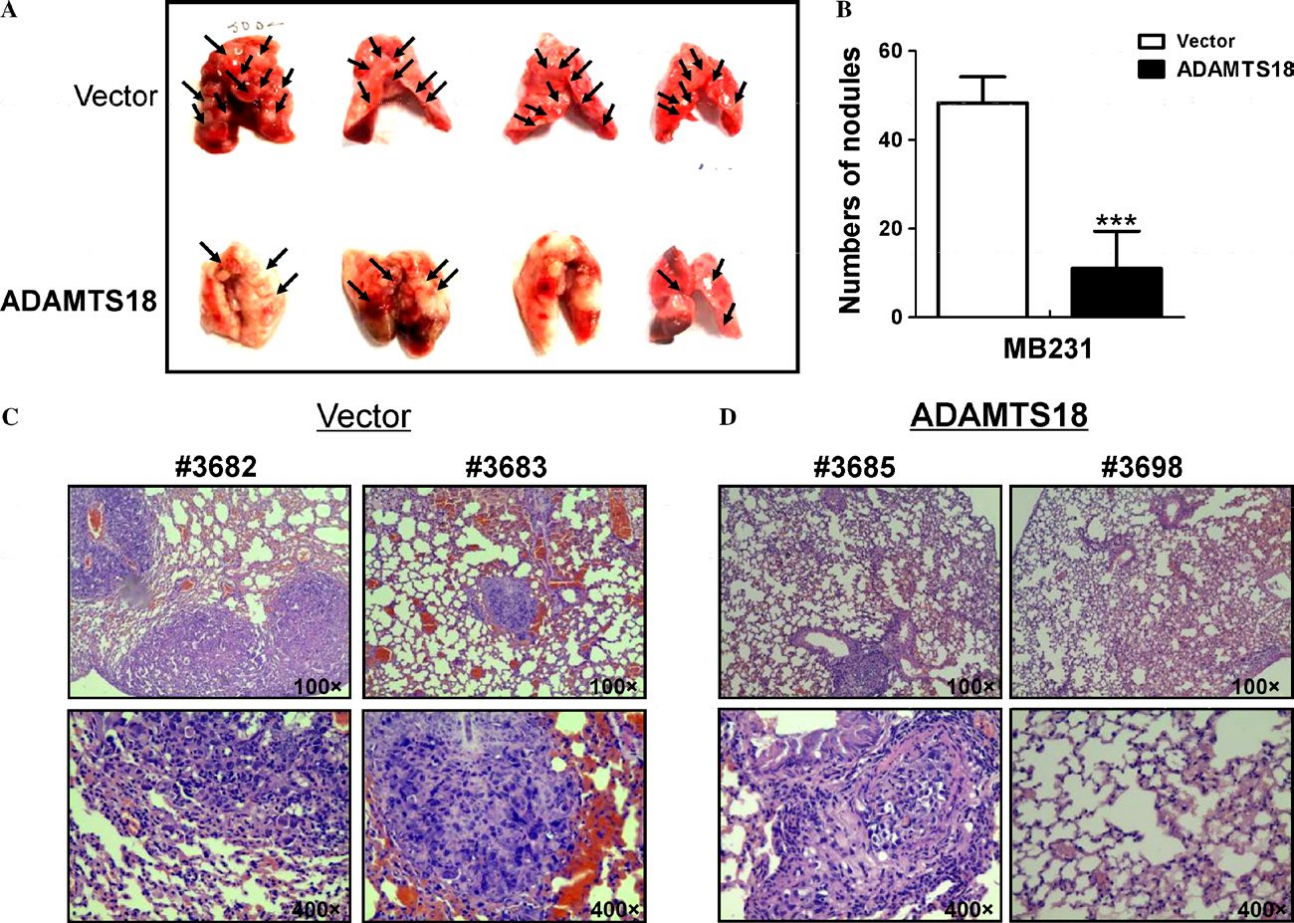
ADAMTS18 inhibits breast cancer cells' metastasis in vivo. (A) Representative images of lungs with metastatic nodules 2 months after injection of control and *ADAMTS18* overexpressed MB231 stable cell lines. Arrows indicate metastatic nodules. (B) The number of lung metastatic nodules was counted under a dissecting microscope. Compared with the control group, a statistically dramatic decrease in the number of the lung metastases was seen in the *ADAMTS18* overexpressing cell group. (C) Hematoxylin and eosin (H&E) staining of sections of lungs 2 months after injection of *ADAMTS18* overexpressing MB231 cells into Balb/c nude mice through their tail veins. Upper panel, magnification, 100×; lower panel, magnification, 400×. *P <* *0.05, **P <* *0.01, and ***P <* *0.001.

To evaluate tumor metastatic ability of *ADAMTS18* in vivo, two groups of the Balb/c nude mice were randomly injected intravenously in the tail vein with *ADAMTS18*‐transfected or vector (Vec)‐transfected MB231 cells. As shown in Figure 4, the *ADAMTS18* overexpression group mice had a significantly small number and sparse lung nodules than the control group (*P *<* *0.001). Hematoxylin and eosin (H&E) staining confirmed that the nodules selected randomly on the surfaces of mice lungs were metastatic tumors. Histological analyses further confirmed that *ADAMTS18* suppresses lung metastasis of MB231 cells in nude mice (Fig. 4C).

### ADAMTS18 suppresses epithelial to mesenchymal transition of breast cancer cells

**Figure 5:**
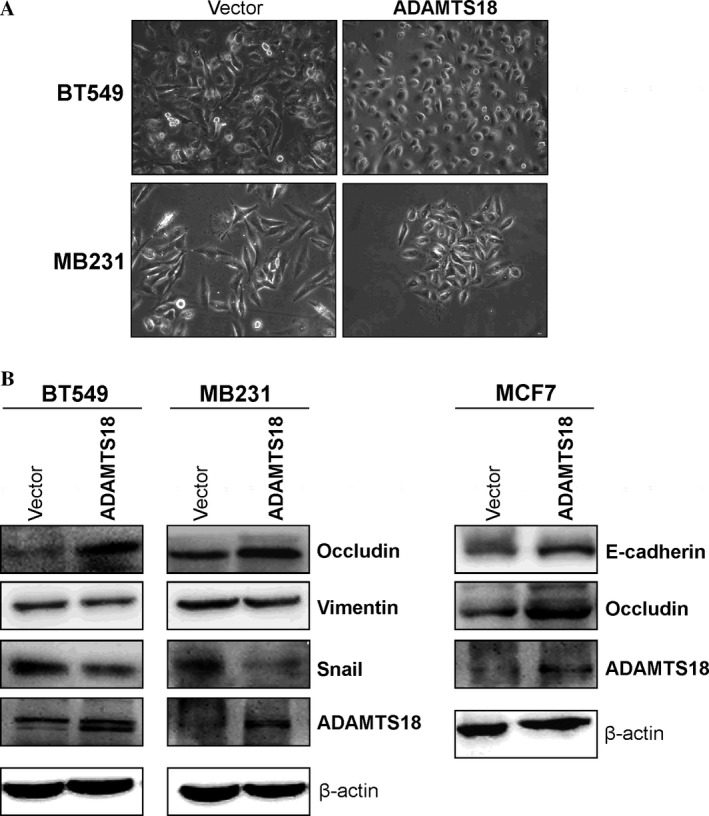
ADAMTS18 inhibited tumor cell EMT. (A) Morphological changes of BT549 and MB231 cells after *ADAMTS18* transfection as seen by phase contrast microscopy. Original magnification, 100×. (B) Western blots showing the expression of E‐cadherin, occludin, vimentin, and Snail in *ADAMTS18* or vector‐transfected cells. *β*‐actin was used as a loading control.

Epithelial to mesenchymal transition (EMT) plays a crucial role in tumor cell metastasis. Cell morphology assay showed cobblestone‐like changes in *ADAMTS18*‐expressing BT549 and MB231 cells (Fig. 5A), suggesting the epithelial phenotype conversion. EMT‐related proteins were further examined. Consistent with the morphological changes, our results showed that vimentin and Snail proteins were decreased and epithelial marker occludin was increased in *ADAMTS18‐*transfected BT549 and MB231 cells compared to the control cells, while E‐cadherin and occludin was increased and Snail was decreased in *ADAMTS18‐*expressing MCF7 cells (Fig. 5B). These data demonstrate that *ADAMTS18* suppresses EMT of breast tumor cells.

### ADAMTS18 suppresses AKT and NF‐kB signaling pathways in breast cancer cells

**Figure 6:**
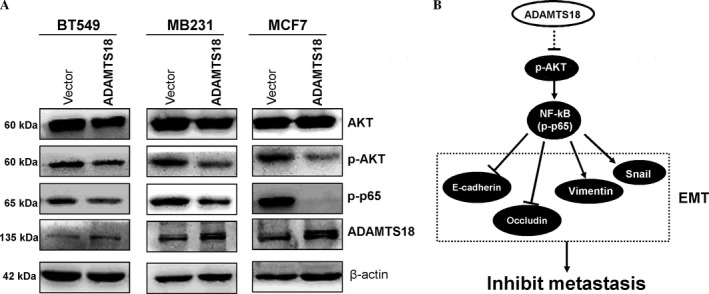
Ectopic expression of ADAMTS18 disrupts AKT and NF‐*κ*B signaling. (A) Western blotting was performed in *ADAMTS18* or vector‐transfected BT549, MB231, and MCF7 cells, using antibodies against AKT, phospho‐AKT, and phospho‐P65. *β*‐actin was used as a control. (B) Proposed mechanism of tumor‐suppressive function of *ADAMTS18* through suppression the AKT and NF‐*κ*B signaling pathway.

To further explore molecular mechanisms responsible for *ADAMTS18‐*mediated EMT suppression, we analyzed the effects on AKT and NF‐kB signaling pathways 21. As expected, *ADAMTS18*‐transfected BT549, MB231, MCF7 cells—all displayed lower levels of phosphorylated AKT and phosphorylated p65 than the control cells (Fig. 6A). These findings suggest that *ADAMTS18* may act as an antagonist of AKT and NF‐KB signaling pathway during cell EMT progression in breast cancer (Fig. 6B).

## Discussion

In this study, we found frequent downregulation or silencing of *ADAMTS18* by promoter methylation in breast cancer cells and primary tissues. *ADAMTS18* is methylated in 70.8% of primary breast cancers, but not in normal breast tissues, indicating tumor‐specific methylation. Demethylation treatments could restore *ADAMTS18* expression in breast cancer cells, indicating that promoter methylation directly mediates its silencing. Our data show that ectopic expression of ADAMTS18 inhibited the migration and invasion of breast cancer cell lines through downregulation of AKT and NF‐*κ*B activity with no significant effect on cell proliferation. ADAMTS18 suppressed tumor metastasis of breast cancer in vivo. These results suggest that ADAMTS18 functions a tumor suppressor through suppressing migration and invasion in breast cancer.

Epigenetic inactivation includes promoter methylation and histone modification, which further regulates cancer gene expression 22, 23. *ADAMTS18* was downregulated and methylated in clear cell renal cell, gastric, colorectal, and pancreatic carcinomas 10, 11. However, another *ADAMTS18* family member, *ADAMTS8* was frequently decreased by promoter methylation in common carcinoma cell lines 24. *ADAMTS9* was reduced by promoter methylation in gastric cancer cells, and *ADAMTS15* was genetically inactivated in colon cancer. Here, we found promoter methylation and downregulation of *ADAMTS18* in primary breast cancer tissues and cell lines. There was no methylation detected in SK‐BR‐3 and YCC‐B3 breast cancer cell lines with silenced *ADAMTS18*, suggesting that other mechanisms like histone modifications mediated *ADAMTS18* silencing. We found that *ADAMTS18* promoter methylation was observed in 34 out of 48 (70.8%) primary breast carcinoma sample. However, previous study 8 presented that *ADAMTS18* methylation in breast carcinoma was 24% (5/21). The reason caused the difference may be different sample sources from Hong Kong and mainland, or limited sample sizes, which needs further investigation by a large sample‐sized study. In our previous study, we also found frequent methylation of ADMTS18 in other common tumors like 70% of NPC, 52% of ESCC, and 63% of cervical cancer 8. Thus, development of quantitative detection method for ADAMTS18 methylation will be our next study.

Although, as demonstrated here and in other studies, *ADAMTS18* is downregulated in multiple cancers, few studies have been reported concerning its related mechanism in tumorigenesis. Our study appears to be the first to reveal its underlying mechanism. We found that the inhibition of tumor migration and invasion by *ADAMTS18* are associated with deregulation of AKT and NF‐*κ*B signaling pathway, in which AKT pathway was in line with the findings in *ADAMTS9*, another member of the same subgroup 6. *ADAMTS9* functions as a tumor suppressor through decreasing cell proliferation and inhibiting angiogenesis via regulating AKT/mTOR pathway.

AKT signaling also plays a crucial role in EMT, further leading to tumor migration and invasion 25. Alternatively, AKT can liberate NF‐*κ*B (p‐P65) to enter into the nucleus and upregulate Snail and Slug expression to promote EMT 26, 27, 28. Further investigation of the exact mechanism by which *ADAMTS18* can act on AKT during the course of multistep tumor progression is needed.

In summary, we demonstrate that *ADAMTS18* silencing in breast cancer is significantly correlated with promoter CpG methylation. ADAMTS18 acts as an antagonist of AKT and NF‐*κ*B signaling, further suppressing EMT and metastasis of breast cancer cells. Our study may help to establish new cancer gene therapeutic strategies for breast cancer.

## Conflicts of Interest

The authors declare no conflict of interest.

## Supporting information


**Table S1**. *ADAMTS18* methylation and clinicopathologic features of breast tumors
**Table S2**. List of primers used in this study
