# Supplementary material for: Epigenetic silencing of ADAMTS18 promotes cell migration and invasion of breast cancer through AKT and NF‐κB signaling
Source: Cancer Med. 2017 May 15;6(6):1399–408. doi: 10.1002/cam4.1076 (PMC5463072; doi:10.1002/cam4.1076)
Supplement: Supplementary file 1 — Table S1. ADAMTS18 methylation and clinicopathologic features of breast tumors Table S2. List of primers used in this study [file CAM4-6-1399-s001.docx]

| **Suppl table 1 *ADAMTS18* methylation and clinicopathologic features of breast tumors** | | | | | |
| --- | --- | --- | --- | --- | --- |
| **Clinicopathologic features** |  | **Number  (n = 48)** | ***ADAMTS18* methylation status** | | ***p* value** |
|  |  |  | **Methylated** | **Unmethylated** |  |
| Age (years) | ≤40 | 6 | 2 | 4 | 0.0557 |
|  | ＞40 | 41 | 31 | 10 |  |
|  | Unknown | 1 | 1 | 0 |  |
| Tumor grade | I | 3 | 3 | 0 | 0.579 |
|  | II | 35 | 24 | 11 |  |
|  | III | 2 | 1 | 1 |  |
|  | Unknown | 8 | 6 | 2 |  |
| Tumor size | ＜2.0 cm | 16 | 10 | 6 | 0.273 |
|  | ≥2.0cm，≤5.0cm | 24 | 20 | 4 |  |
|  | > 5.0 cm | 5 | 3 | 2 |  |
|  | Unknown | 3 | 1 | 2 |  |
| Lymph node metastasis | Positive | 29 | 19 | 10 | 0.4884 |
|  | Negative | 15 | 12 | 3 |  |
|  | Unknown | 4 | 3 | 1 |  |
| Distant metastasis | Positive | 2 | 2 | 0 | 1 |
|  | Negative | 43 | 31 | 12 |  |
|  | Unknown | 3 | 1 | 2 |  |
| ER status | Positive | 27 | 17 | 10 | 0.165 |
|  | Negative | 14 | 12 | 2 |  |
|  | Unknown | 7 | 5 | 2 |  |
| PR status | Positive | 21 | 12 | 9 | 0.0888 |
|  | Negative | 19 | 16 | 3 |  |
|  | Unknown | 8 | 6 | 2 |  |
| HER2 status | Positive | 33 | 25 | 8 | 0.2024 |
|  | Negative | 8 | 4 | 4 |  |
|  | Unknown | 7 | 5 | 2 |  |
| P53 status | Positive | 24 | 18 | 6 | 0.4898 |
|  | Negative | 16 | 10 | 6 |  |
|  | Unknown | 8 | 6 | 2 |  |
| Ki67 status | >14% | 25 | 19 | 6 | 0.311 |
|  | <14% | 15 | 9 | 6 |  |
|  | Unknown | 8 | 6 | 2 |  |

**Suppl table 2 List of primers used in this study**

| **RT-PCR** | **Primer** | **Sequence(5’-3’)** | **Product size (bp)** |
| --- | --- | --- | --- |
|  | ADAMTS18-F | TAGCCAGTGACAGCAGCAG | 195 |
|  | ADAMTS18-R | CTAAGTGCAGTTCCTGTCCA |  |
|  | β-actin-F | CCTGTGGCATCCACGAAACT | 314 |
|  | β-actin-R | GAAGCATTTGCGGTGGACGAT |  |
| **MSP** |  |  |  |
|  | ADAMTS18m1 | TTGTAGTTCGGTAGGTTCGC | 114 |
|  | ADAMTS18m2 | ACTCCAAATAAAAACCGCCG |  |
|  | ADAMTS18u1 | AAATTGTAGTTTGGTAGGTTTGT | 119 |
|  | ADAMTS18u2 | CAACTCCAAATAAAAACCACCA |  |
